# Tennis Players and Water Polo Athletes Now Have Something in Common to Talk About: MRI Findings of Extensor Carpi Ulnaris Chronic Subsheath Injury

**DOI:** 10.7759/cureus.2489

**Published:** 2018-04-16

**Authors:** Nishant Gupta, Neeraj Bhatt, Itisha Bansal, Shuo Li, Yogesh Kumar

**Affiliations:** 1 Radiology, Columbia University Medical Center; 2 Department of Radiology, Yale New Haven Health at Bridgeport Hospital, Bridgeport, USA; 3 Anesthesiology, Newyork-presbyterian Brooklyn Methodist Hospital, Newyork, USA

**Keywords:** chronic subsheath injury, extensor carpi ulnaris tendon dislocation, us, mri, chronic wrist injury

## Abstract

Pathologies of the extensor carpi ulnaris (ECU) tendon are often due to de Quervain's tenosynovitis of the first dorsal compartment among the wrist tendon pathologies. A common cause for tendinitis and tenosynovitis of the ECU tendon is its dislocation. ECU dislocation is unique among all wrist tendon injuries due to its typical location within a fibro-osseous tunnel bordered by a fibrous sheath, which is termed as the subsheath. The subsheath is the main anatomic structure keeping the ECU tendon within the tunnel. Subsheath tears can lead to a fixed or dynamic pattern of ECU dislocation. This injury is more often seen in tennis players and golfers than in water polo athletes, as there are overall fewer water polo athletes when compared to tennis players and golfers. In this article, we will discuss the mechanisms, clinical presentation, magnetic resonance imaging (MRI) findings, differential diagnoses, and management options for chronic subsheath tears.

## Introduction

Extensor carpi ulnaris (ECU) tendon abnormalities are often due to de Quervain’s tenosynovitis of the first dorsal compartment among the wrist tendon pathologies [1]. The ECU tendon is uniquely secured within a distal ulnar groove, also called the fibro-osseous tunnel, by a fibrous sheath also called the subsheath [2-3]. A tear in this fibrous sheath due to forceful forearm supination results in ECU tendon dislocation outside the distal ulnar groove. The dislocation may be fixed or dynamic. This injury pattern is seen in sports like tennis, golf, and water polo, which utilize active wrist movements. In this article, we will discuss the key magnetic resonance imaging (MRI) features of subsheath tears and ECU tendon dislocation.

## Case presentation

**Figure 1:**
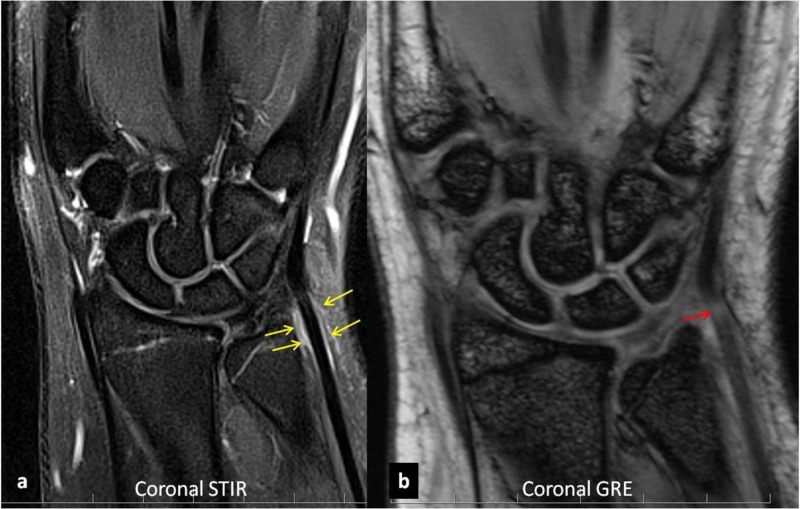
Coronal STIR (a) of the right wrist shows fluid signal along the ulnar side of the wrist, around the ECU tendon (yellow arrows). Coronal GRE (b) shows altered signal in the ECU tendon substance (red arrow). STIR: short tau inversion recovery; ECU: extensor carpi ulnaris; GRE: gradient echo imaging

The patient was a 14-year-old active water polo player, who mainly played at offensive positions, mainly the center forward position. She had also been a very active tennis player four years ago, but she had switched to water polo due to her interest in the sport. She started complaining of ulnar-sided right wrist pain two years ago, especially after long water polo practice sessions and matches. She faintly remembered similar but less intense pain while playing tennis four years ago. Due to her ongoing ulnar pain, she underwent wrist radiographs, which were normal. Due to her intermittent ulnar-sided right wrist pain for the past three years, with weakness in the wrist and hand, there was a clinical concern of a triangular fibrocartilage complex (TFCC) tear. She underwent right wrist MRI which showed no triangular fibrocartilage tear or other internal derangements. Fluid signal was noted around the ECU tendon (Figure 1).

**Figure 2:**
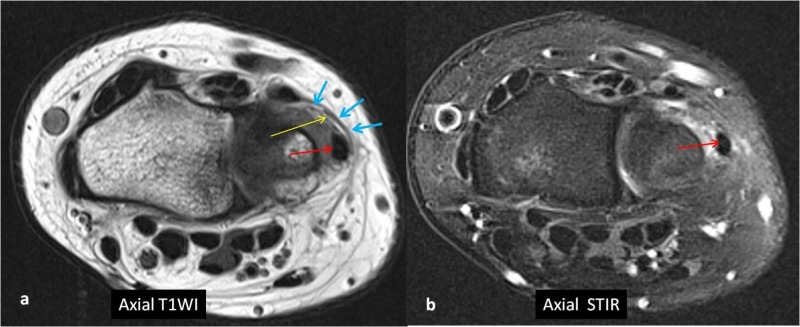
Axial T1WI (a) and axial STIR (b) at the level of the distal ulna shows fluid around the ECU tendon (red arrow). The ECU tendon is slightly more palmar than normal and is outside the ulnar groove. The subsheath of the ECU shows diffuse fragmentation (yellow arrow). Blue arrows indicate the overlying extensor retinaculum T1W1: T1 weighted image; STIR: short tau inversion recovery; ECU: extensor carpi ulnaris

The ECU tendon was subluxed out of the ulnar groove due to a subsheath tear (Figure 2). She was treated with wrist immobilization in a cast in extension and slight radial deviation for eight weeks. This position helps place the ECU tendon in the ulnar groove. The patient resumed water polo after 10 weeks and is currently doing well.

## Discussion

**Figure 3:**
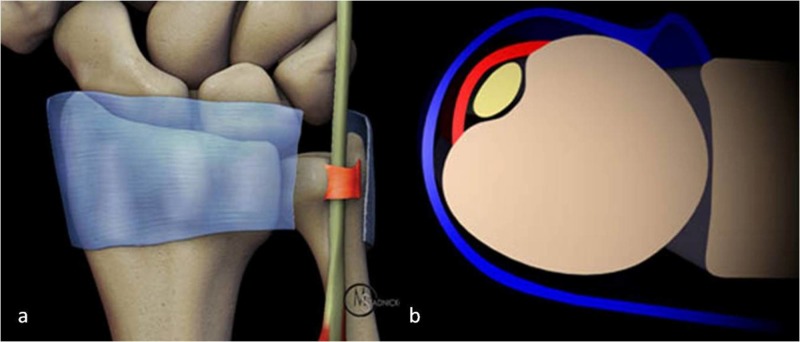
Artistic illustration of the ECU at the distal ulnar level (a) shows cut extensor retinaculum (blue). The ECU tendon is stabilized by a band-like subsheath (red) in the ulnar groove. Graphic axial representation (b) shows extensor retinaculum (blue) over the ECU tendon. Note the ECU subsheath (red). ECU: extensor carpi ulnaris Image used with permission from MRI Web Clinic - Radsource.us.

The ECU tendon is a component of the sixth extensor compartment of the wrist. The ECU muscle originates from the lateral epicondyle of humerus and runs along the middle third of the dorsal surface of ulna. It inserts at the base of the fifth metacarpal [2-4]. It is kept secure in the distal ulnar groove in a fibro-osseous tunnel by a fibrous sheath deep to the extensor retinaculum as described by Spinner and Kaplan, termed as the subsheath. The subsheath acts as the chief anatomical restraint and keeps the ECU tendon within the groove (Figure 3). The extensor retinaculum does not play any pivotal role in this regard. The mechanism of the subsheath injury is a combination of forceful supination of the forearm along with ulnar deviation and palmar flexion of the wrist, which is a common action in tennis, golf, as well as water polo [5-7]. As expected, loss of integrity of the subsheath results in the ECU tendon moving out of its place from the ulnar grove whenever the tendon is taut, such as forearm supination, ulnar deviation, and palmar flexion of the wrist.

Clinically, there may or may not be a reproducible history of acute injury. The patient may rarely mention a popping sensation on the ulnar aspect of the wrist at the time of trauma. On examination, there is tenderness and swelling on the ulnar aspect of the wrist. Provocative maneuvers such as supination, palmar flexion, and ulnar deviation will elicit or worsen the pain. These maneuvers may elicit palpation of the dislocated ECU tendon, which is otherwise not elicited in the neutral position [4, 8].

Plain radiography is insensitive to detect this entity, as in this case. Ultrasound imaging is more effective for detecting ECU tendon dislocation with the added advantage of providing real-time imaging to detect a dynamic dislocation. Direct sonographic visualization of a subsheath tear is quite unlikely, but the tendon dislocation in itself may act as a surrogate marker of the subsheath tear. The advantage of MRI is direct visualization of a subsheath tear. However, in comparison to ultrasound, the limitation of MRI is its lack of dynamic imaging. Many times, the wrist is placed in the pronated position while taking the MRI, and if the ECU subluxation is not fixed but dynamic, it may be in its native position within the groove and a careful evaluation to look for secondary signs is required [9-10].

**Figure 4:**
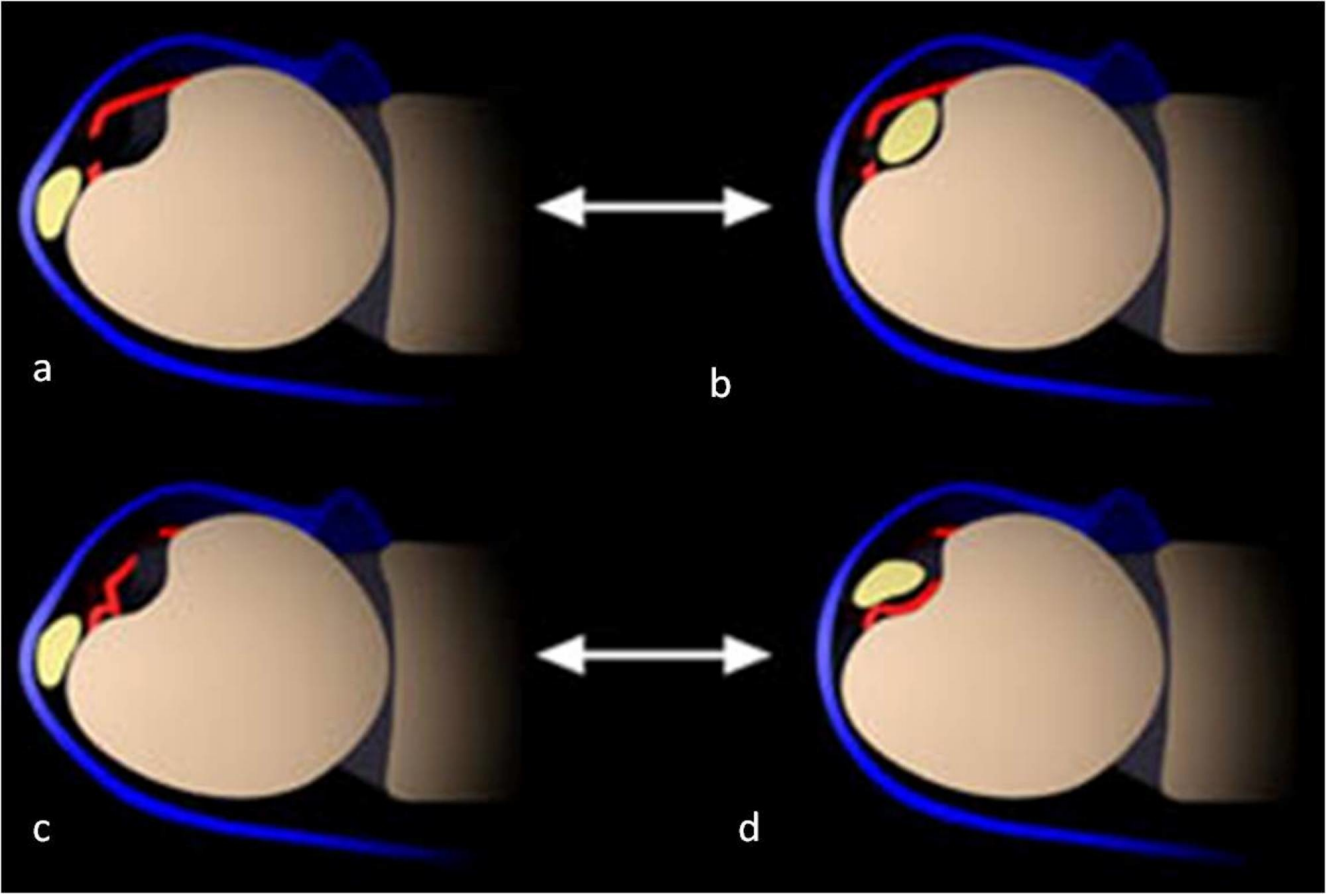
Schematic representation of a rupture of the ECU subsheath; (a, b) show tear along the ulnar side of the subsheath. Ulnar-sided tears lead to dislocation of the ECU tendon transiently, with relocation on pronation as shown in image b. A radial-sided tear is shown in images c and d, in which the dislocated tendon remains on top of the torn subsheath even after relocation. ECU: extensor carpi ulnaris Image used with permission from MRI Web Clinic - radsource.us.

Inoue and Tamura published a frequently cited study on subsheath tears and ECU dislocation. They classified it into three types [11]. In type A, the sheath is damaged on the ulnar side, and the torn portion of the sheath lies superficial to the ECU tendon as the tendon relocates back to its native position in the distal ulnar groove through the defect (Figures 4a-4b). Type B is characterized by a tear in the sheath on the radial side. Though the tendon lies in the groove on pronation, it sits above the torn sheath (Figure 4c-4d). In type C, there is no focal rent through the sheath, but there is avulsion of the ulnar side of the periosteum in continuity with the sheath, forming a pouch in which tendon dislocates.

The management varies from conservative cast immobilization for six weeks with the wrist in pronation to surgical repair. Roland first reported a case of acute sheath tear with ECU subluxation; perioperatively, the tendon retracted back within the tunnel on pronation. However, the gap between the torn ends of the sheath remained wide enough to necessitate surgical repair. He argued that the wide gap between the torn ends necessitated surgery as against conventional conservative management of six weeks of wrist immobilization in the pronated position [8].

If surgical management is warranted, it may vary depending upon the tear type as proposed by Inoue and Tamura. In type A tears, the sheath is repaired using a strip of the extensor retinaculum. Type B tears are repaired by direct suturing the free edge of the torn sheath with the soft tissues on the radial aspect. In Type C, the sheath is incised. The tendon is brought back to its anatomical position in the groove, and the sheath is sutured over it. The periosteum is drilled back to the bone [11].

Any kind of surgical management is followed by keeping the wrist in immobilization using a cast in 90 degrees flexion and in a neutral rotation position for six weeks. This is followed by restriction of heavy activities for an additional three months.

It is to be noted that type B injuries are not amenable to conservative management using cast immobilization in pronation, as the tendon overlying the torn sheath will preclude healing. It is also not a good alternative for type 1 Injuries if there is a large gap between the torn edges of the sheath as stated earlier by Roland.

Causes of ulnar pain due to ECU abnormalities other than its dislocation due to subsheath tears are isolated tendinopathy and tendon rupture. Other causes of ulnar side pain are the injury of other components of the triangular fibrocartilage complex (TFCC), tear of the triquetrum-lunate ligament, and carpal fractures such as in the hook of hamate, lunate, and triquetrum [5].

## Conclusions

An unrecognized ECU dislocation and underlying subsheath tear may result in chronic abnormalities at the distal radioulnar joint. Appropriately recognizing the subsheath tear will help in choosing between conservative and operative management. It can also serve as a guide to decide among the types of surgery best suited for individual cases. The radiologists should be aware of this not so uncommon entity. Under the clinical settings of distal ulnar pain brought on by certain maneuvers, ECU dislocation and tendon abnormalities should be actively considered. All attempts should be made to identify the subsheath tear and characterize it.
